# Beneficial effect of temporary methotrexate interruption on B and T cell responses upon SARS-CoV-2 vaccination in patients with rheumatoid arthritis or psoriatic arthritis

**DOI:** 10.1038/s41541-024-00805-3

**Published:** 2024-01-30

**Authors:** Pedro Martínez-Fleta, Esther F. Vicente-Rabaneda, Ana Triguero-Martínez, Emilia Roy-Vallejo, Miren Uriarte-Ecenarro, Francisco Gutiérrez-Rodríguez, Patricia Quiroga-Colina, Ana Romero-Robles, Nuria Montes, Noelia García-Castañeda, Gina P. Mejía-Abril, Jesús A. García-Vadillo, Irene Llorente-Cubas, José R. Villagrasa, José M. Serra López-Matencio, Julio Ancochea, Ana Urzainqui, Laura Esparcia-Pinedo, Arantzazu Alfranca, Hortensia de la Fuente, Rosario García-Vicuña, Francisco Sánchez-Madrid, Isidoro González-Álvaro, Santos Castañeda

**Affiliations:** 1 Department of Immunology, Hospital Universitario de La Princesa IIS-Princesa (Instituto de Investigación Sanitaria del Hospital Universitario de La Princesa), Madrid, Spain; 2 Department of Rheumatology, Hospital Universitario de La Princesa IIS-Princesa (Instituto de Investigación Sanitaria del Hospital Universitario de La Princesa), Madrid, Spain; 3 Department of Internal Medicine, Hospital Universitario de La Princesa IIS-Princesa (Instituto de Investigación Sanitaria del Hospital Universitario de La Princesa), Madrid, Spain; 4 Clinical Pharmacology Department, Hospital Universitario de La Princesa, Instituto Teófilo Hernando, Instituto de Investigación Sanitaria La Princesa (IP), Universidad Autónoma de Madrid (UAM), Madrid, Spain; 5 Department of Medicine, Universidad Autónoma de Madrid (UAM), Madrid, Spain; 6 Department of Preventive Medicine, Hospital Universitario de La Princesa IIS-Princesa (Instituto de Investigación Sanitaria del Hospital Universitario de La Princesa), Madrid, Spain; 7 Department of Hospital Pharmacy, Hospital Universitario de La Princesa IIS-Princesa (Instituto de Investigación Sanitaria del Hospital Universitario de La Princesa), Madrid, Spain; 8 Department of Pneumology, Hospital Universitario de La Princesa IIS-Princesa (Instituto de Investigación Sanitaria del Hospital Universitario de La Princesa), Madrid, Spain; 9 Cátedra UAM-Roche, EPID-Future, Department of Medicine, Universidad Autónoma de Madrid (UAM), Madrid, Spain; 10 Centro de Investigación en Red de Enfermedades Respiratorias (CIBERES), Instituto de Salud Carlos III (ISCIII), Madrid, Spain; 11 CIBER Cardiovascular CIBERCV, Madrid, Spain

**Keywords:** Translational research, RNA vaccines

## Abstract

B and T cell responses were evaluated in patients with rheumatoid arthritis (RA) or psoriatic arthritis (PsA) after 1 or 2 weeks of methotrexate (MTX) withdrawal following each COVID-19 vaccine dose and compared with those who maintained MTX. Adult RA and PsA patients treated with MTX were recruited and randomly assigned to 3 groups: MTX-maintenance (*n* = 72), MTX-withdrawal for 1 week (*n* = 71) or MTX-withdrawal for 2 weeks (*n* = 73). Specific antibodies to several SARS-CoV-2 antigens and interferon (IFN)-γ and interleukin (IL)-21 responses were assessed. MTX withdrawal in patients without previous COVID-19 was associated with higher levels of anti-RBD IgG and neutralising antibodies, especially in the 2-week withdrawal group and with higher IFN-γ secretion upon stimulation with pools of SARS-CoV-2 S peptides. No increment of RA/PsA relapses was detected across groups. Our data indicate that two-week MTX interruption following COVID-19 vaccination in patients with RA or PsA improves humoral and cellular immune responses.

## Introduction

Vaccination has proven to be the main strategy to attenuate severe symptoms leading to hospitalisation due to COVID-19 in the general population. mRNA vaccines have prevailed in most countries due to their safety, high immunogenicity, low cost, and their quick and easy manufacture^1,2^. However, at the time of recruiting participants for this study (March through September 2021, during first vaccination campaign in Spain) there were few data addressing the immunogenicity of COVID-19 vaccines in immunocompromised individuals, such as patients with immune-mediated inflammatory diseases (IMID). Furthermore, at the beginning of the pandemic initial data suggested that patients with rheumatoid arthritis (RA), among other IMID, may be more susceptible to developing severe COVID-19, leading to hospitalisation or intensive care unit admission, although later evidence revealed the great importance of other factors such as advanced age and comorbidities^3–7^.

RA patients are usually treated with disease-modifying anti-rheumatic drugs (DMARDs) such as methotrexate (MTX), either in monotherapy or in combination with other conventional synthetic (csDMARDs), biologic (bDMARDs) or targeted synthetic (tsDMARDs) DMARDs. Hence, the humoral response to vaccination might be impaired due to these immunomodulatory treatments, as it has been previously described for influenza and pneumococcal vaccines^8^. Additionally, it has been hypothesised that DMARDs could have a negative effect on T cell responses upon pneumococcal vaccination^9^. Accordingly, functional in vitro studies are necessary to try to unravel the role of these immunomodulatory drugs on vaccine immunogenicity.

Temporary MTX discontinuation has been proposed as a possible strategy to strengthen the antibody response after vaccination^10^. In fact, two prospective randomised studies have evaluated the effect of a temporary MTX interruption on the immune response in patients with RA vaccinated against influenza, showing that a 2-week MTX discontinuation was safe (no increase in disease activity was observed upon discontinuation) and had a beneficial impact on humoral response^11,12^. A short interruption of MTX after COVID-19 vaccine doses has also proven to be effective to improve antibody response in these patients^13–17^. However, few data regarding T cell response after MTX interruption are available. In this context, we postulated that temporary MTX interruption after COVID-19 vaccine administration could be beneficial for RA and psoriatic arthritis (PsA) patients in terms of improving both humoral and cellular response against SARS-CoV-2. To test this hypothesis, we designed a prospective observational study in which adult patients with RA and PsA were recruited and randomised into 3 groups: one group maintaining MTX treatment unchanged and two additional groups discontinuing MTX for 1 or 2 weeks after vaccination. We assessed antibody response through detection of specific IgG, IgA and IgM against different SARS-CoV-2 antigens and cellular response by measuring secretion of the antiviral cytokine interferon (IFN)-γ as well as interleukin (IL)-21, because of its importance in T cell cytotoxic activity and B cell activation^18–20^.

## Results

### Demographic and clinical features of the study populations

**Fig. 1:**
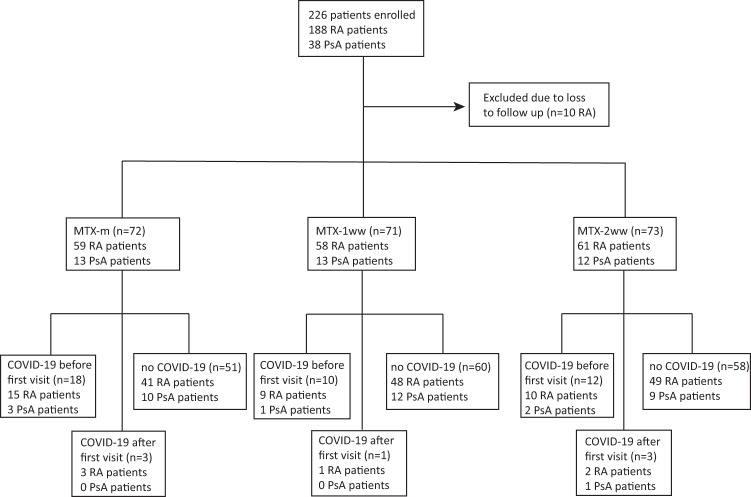
Flow chart of the study population. Distribution of the patients into the three study groups: MTX-maintaining group (MTX-m), one week of MTX withdrawal (MTX-1ww) and two week of MTX withdrawal (MTX-2ww). Previous history of SARS-CoV-2 infection was determined for all patients by means of serology.

A total of 226 patients with RA or PsA fulfilled inclusion criteria and were recruited for the study, although 10 patients were lost to follow-up and hence discarded. The final study population consisted of 216 patients (178 RA and 38 PsA) distributed in three groups: the first group maintained MTX treatment unchanged during vaccination (MTX-m) (*n* = 72), while the second (MTX-1ww) (*n* = 71) and third (MTX-2ww) (*n* = 73) groups withdrew MTX for 1 or 2 weeks after each vaccine dose, respectively (Fig. 1). The median age of the whole study population was 56 years (IQR: 47-67) and more than 80% of the participants were women. The majority of the patients (71%) received the BNT162b2 mRNA vaccine, whereas 14% were vaccinated with ChadOX-1-S and only 6% and 9% received mRNA-1273 and Ad26.COV2.S, respectively. No significant differences in age, sex, comorbidities, MTX dose, use of other DMARDs, lymphocyte count, days after vaccination or type of administered vaccine were observed across groups (Table 1).

**Table 1:** Demographic and clinical characteristics of the study population.

	MTX-m	MTX-1ww	MTX-2ww	
	*n* = 72	*n* = 71	*n* = 73	*p*-value
Age	57 (48–63)	57 (45–67)	56 (47–69)	0.79
Sex (female)	57 (79.2)	58 (81.7)	58 (79.5)	0.92
Ethnicity (White)^1^	64 (88.9)	63 (88.7)	57 (78.1)	0.24
Comorbidities^3^	46 (63.9)	35 (49.3)	46 (63)	0.14
Rheumatoid arthritis	59 (81.9)	58 (81.7)	61 (83.6)	0.13
Psoriatic Arthritis	13 (18.1)	13 (18.3)	12 (16.4)	0.95
MTX dose (mg/week)	15 (10–17.5)	15 (10–15)	15 (10–17.5)	0.71
DAS28-CRP^2^ [0–8.47]	2.3 (1.7–3.2)	2.1 (1.5-3)	2.3 (1.6–3.1)	0.58
CRP (mg/dL) [0–0.5]	0.2 (0.1–0.4)	0.2 (0.1–0.3)	0.2 (0.1–0.5)	0.12
Lymphocyte count (cells/mm^3^) [1.5 × 10^3^-4.0 × 10^3^]	1865 (1465–2440)	2020 (1520–2440)	2010 (1595–2315)	0.70
Biologic DMARDs	19 (26.4)	22 (31)	26 (35.6)	0.49
TNF inhibitors	16 (22.2)	20 (28.2)	19 (26)	—
IL-6 inhibitors	2 (2.8)	2 (2.8)	5 (6.8)	—
IL-17 inhibitors	1 (1.4)	1 (1.4)	1 (1.4)	—
Rituximab	0 (0)	0 (0)	1 (1.4)	—
Other csDMARDs	12 (16.7)	12 (16.9)	13 (17.8)	0.95
Hydroxycloroquine	5 (6.9)	3 (4.2)	3 (4.1)	—
Sulphasalazine	3 (4.2)	4 (5.6)	3 (4.1)	—
Leflunomide	4 (5.6)	5 (7)	7 (9.9)	—
tsDMARDs	4 (5.6)	3 (4.2)	2 (2.7)	0.65
Tofacitinib	0 (0)	2 (2.8)	0 (0)	—
Baricitinib	4 (5.6)	1 (1.4)	1 (1.4)	—
Upadacitinib	0 (0)	0 (0)	1 (1.4)	—
Glucocorticoids	7 (9.7)	9 (12.7)	13 (17.8)	0.40
Average dose of GC (mg/day)	5 (2.5-10)	3.8 (2.5-6.2)	3.8 (2.5-5)	0.70
Type of vaccine				0.15
One dose of BNT162b2	4 (5.6)	3 (4.2)	9 (12.3)	—
Two doses of BNT162b2	47 (65.3)	44 (62)	46 (63)	—
One dose of ChAdOx-1-S	1 (1.4)	0 (0)	0 (0)	—
Two doses of ChAdOx-1-S	11 (15.3)	13 (18.3)	5 (6.8)	—
One dose of mRNA-1273	0 (0)	1 (1.4)	1 (1.4)	—
Two doses of mRNA-1273	5 (6.9)	1 (1.4)	6 (8.2)	—
One dose of Ad26.COV2.S	4 (5.6)	9 (12.7)	6 (8.2)	—
Days between vaccine doses^4^	21 (21–115)	21 (21–96)	21 (21–90)	0.91
Days from vaccination completion to blood sampling	28 (28–35)	29 (28–35)	29 (28–35)	0.85
COVID-19 pre-vaccination	18 (25)	10 (14.1)	12 (16.4)	0.21
COVID-19 after 1^st^ dose	3 (4.2)	1 (1.4)	3 (4.1)	—

^1^Other ethnicities included: Latin American, North African and Black. ^2^DAS28 score is an objective measure of disease activity: <2.6 remission; 2.6–3.2 low disease activity; 3.2–5.1 moderate disease activity; >5.1 high disease activity. ^3^Comorbidities studied: systemic arterial hypertension, diabetes, dyslipidemia, obesity, cardiovascular disease, thromboembolic disease, renal failure, chronic bronchitis, asthma and cancer. ^4^(Min to max range)

All categorical variables are expressed as absolute count (percentage) and quantitative variables as median (IQR). Statistical significance was determined by using ANOVA or Kruskal Wallis for quantitative and chi-square or Fisher exact test for qualitative variables.

*MTX* methotrexate, *DAS28* disease activity score in 28 joints, *CRP* C-reactive protein, *DMARDs* disease-modifying antirheumatic drugs, *csDMARDs* conventional synthetic DMARDs, *tsDMARDs* targeted synthetic DMARDs, *GC* glucocorticoids, *COVID-19* coronavirus disease 2019, *RA* rheumatoid arthritis, *PsA* psoriatic arthritis.

In order to determine the number of subjects previously infected with SARS-CoV-2, we assessed serum specific antibody levels against spike (S), receptor binding domain (RBD) and other antigens not included in the vaccine composition: main protease (Mpro) and nucleoprotein (NP). A total of 47 patients had seroconverted prior to vaccination or before post-vaccine sample collection (18 of them asymptomatic). The number of participants with positive serology for SARS-CoV-2 in the baseline blood sample was higher in the MTX-m group (18) compared with MTX-1ww (10) and MTX-2ww (12) (*p* = 0.13). Besides, seven subjects without previous COVID-19 at baseline had positive serology for Mpro/NP in the final blood sample, hence indicating SARS-CoV-2 infection between first and second visits: 3 belonging to MTX-m group, 1 to MTX-1ww and 3 to MTX-2ww (Fig. 1).

### SARS-CoV-2 vaccination leads to high seroconversion rates in patients with RA/PsA

On average, our data show that COVID-19 vaccination elicited good levels of anti-S IgG, which were higher in patients previously infected with SARS-CoV-2 (Supplementary Fig. 1A). Multivariable analyses performed with standardized variables showed that vaccination exerted a higher effect than previous SARS-CoV-2 infection, probably due to the fact that sample collection was performed closer to the time of vaccination than to infection, although there exist other factors to take into account such as viral inoculum or severity of previous infection. Considering BNT162b2 as the reference condition, ChadOx-1-S and Ad26.COV2.S induced a significantly lower antibody response, whereas mRNA-1273 elicited similar levels of SARS-CoV-2 specific antibodies (Supplementary Fig. 1B). In addition, due to a significant inter-plate variability, specific antibody responses will be presented hereinafter as estimated variables adjusted by confounders (see Supplementary Tables 1 to 2).

### Temporary methotrexate withdrawal is associated with higher antibody responses in patients without previous SARS-CoV-2 infection

**Fig. 2:**
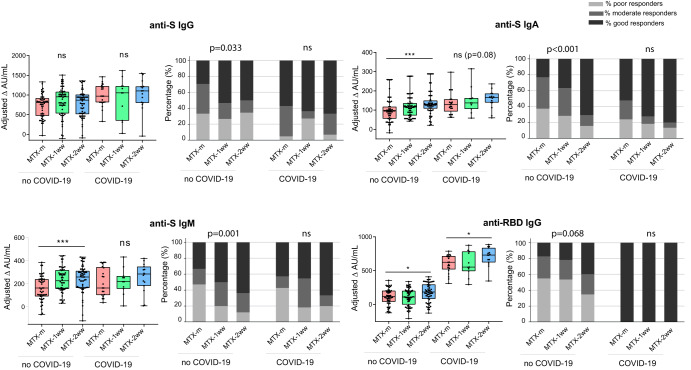
Antibody response after vaccination. Antibody response after SARS-CoV-2 vaccination in MTX-m (*n* = 72), MTX-1ww (*n* = 71) and MTX-2ww (*n* = 73) groups separated in previously uninfected (no COVID-19) and previously infected (COVID-19) individuals. Results are represented as increment (Δ) of AU/mL between pre-vaccination and post-vaccination samples (1 month after complete vaccination), after adjusting for confounding variables by means of glm (left). Cuzick test was used to assess statistical significance. *p < 0.05, ***p < 0.001. Percentage of poor (below 25^th^ percentile response), moderate (25^th^ to 50^th^ percentile) and good responders (greater than 50^th^ percentile) to vaccination across groups (right). Fisher exact test was used to check statistical significance. Box plots represent the interquartile ranges, horizontal lines indicate the medians, and error bars extend to the minimum and maximum observed values.

Seroconversion was widely achieved after vaccination in all the study groups, since anti-S and anti-RBD IgG antibodies were detected in most individuals (Supplementary Fig. 2a, Supplementary Table 3). Interestingly, a significantly higher number of good responders for anti-S IgG were found in both MTX-1ww and MTX-2ww groups compared to the MTX-m group among those previously uninfected (OR 2.7 95%CI [1.2–6.5], OR 2.4 95%CI [1–5.8], *p* = 0.033) (Fig. 2, Supplementary Table 3). Moreover, a significantly higher increment (difference) in arbitrary units/mL (AU/mL) after vaccination was observed in both MTX-ww groups compared to MTX-m group for S-specific IgA and IgM in previously uninfected patients (difference 18.3 95%CI [-4.4–41.1] for MTX-1ww; difference 37.195% CI [13.2–61] for MTX-2ww IgA; difference 69.2 95%CI [29.4–108.9] for MTX-1ww; difference 78.6 95%CI [37.1–120.2] for MTX-2ww IgM; *p* < 0.001). Higher levels of anti-RBD IgG were detected in the MTX-2ww group in individuals with and without prior COVID-19 (difference 106.2 95%CI [18.2–194.2], difference 61.6 95%CI [13.9–109.4], *p* = 0.01; *p* = 0.03) (Fig. 2). Although the majority of the participants had undetectable or in the limit of detection levels of anti-RBD IgA and IgM, we found a trend to higher concentration of antibodies in both MTX-ww groups among patients without previous SARS-CoV-2 infection (Supplementary Fig. 3).

Next, we further investigated whether participants belonging to MTX-ww groups also had higher capacity of virus neutralisation. For that purpose, assays to measure neutralising antibodies (Nabs) in serum were performed, revealing high titres of Nabs in a high proportion of subjects from all groups (Supplementary Table 3). Only 7% of individuals in MTX-m and MTX-1ww groups and 11% in MTX-2ww group failed to produce detectable levels of Nabs (Supplementary Table 3).

**Fig. 3:**
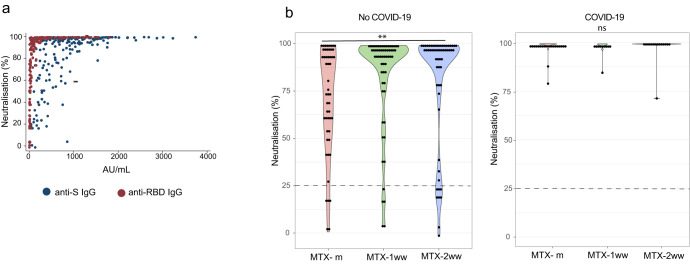
Neutralisation capacity of antibodies after vaccine administration. **a** Scatter plot showing anti-S IgG, anti-RBD IgG and percentage of neutralisation after vaccination in the whole study population (*n* = 216). **b** Percentage of neutralisation after vaccination in MTX-m, MTX-1ww and MTX-2ww. Positivity cut-off is represented as a dotted line. Cuzick test was used to assess statistical significance. **p < 0.01.

We found that a slight increase in anti-RBD IgG was associated with high neutralisation capacity. By contrast, a good response of S-specific IgG didn’t necessarily imply higher levels of Nabs (Fig. 3a). In accordance with the effect observed for specific-RBD IgG, a significantly higher amount of Nabs was observed in both MTX-ww groups versus MTX-m patients in previously uninfected individuals (difference 11.2 95%CI [0.96–21.5] for MTX-1ww, difference 10.3 95%CI [-0.7–21.2]; *p* = 0.004) (Fig. 3b).

### IFN-γ cellular response improves after temporary MTX withdrawal

**Fig. 4:**
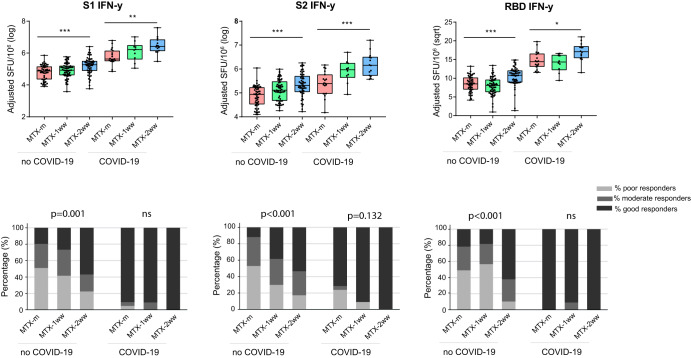
Cellular response after vaccination. IFN-γ response after vaccination in MTX-m (*n* = 66), MTX-1ww (*n* = 68) and MTX-2ww (*n* = 69) separated in previously uninfected (no COVID-19) and previously infected (COVID-19) individuals, after adjusting for confounding variables by means of glm. PBMCs were stimulated with pools of peptides spanning S1, S2 and RBD. Cuzick test was used to assess statistical significance. **p < 0.01 (upper panels). Percentage of poor (below 25^th^ percentile response), moderate (25^th^ to 50^th^ percentile) and good responders (greater than 50^th^ percentile) to vaccination across groups. Fisher exact test was used check statistical significance (lower panels). Box plots represent the interquartile ranges, horizontal lines indicate the medians, and error bars extend to the minimum and maximum observed values.

Multivariable analyses revealed a significant effect of other confounding variables such as the peptide batch; hence, data are shown after adjusting for this confounder (Supplementary Tables 4 and 5). A greater IFN-γ response after stimulating with S1, S2 and RBD pools was found in previously uninfected MTX-ww groups (*p* < 0.001), especially in MTX-2ww group (difference 0.4 [log] 95%CI [0.2-0.6] for S1, difference 0.5 [log] 95%CI [0.3–0.7] for S2, difference 2 [sqrt] 95%CI [1.1–2.9] for RBD) (Fig. 4, Supplementary Fig. 2b). We also observed a higher proportion of IFN-γ good responders upon stimulation with S1, S2 and RBD in MTX-ww groups compared to MTX-m group, in particular upon stimulation with S2 peptides (OR 2.1 95%CI [0.9–5.2] for MTX-1ww, OR 2.5 95%CI [1–6] for MTX-2ww; *p* < 0.001). Remarkably, a higher response for IFN-γ was also detected in MTX-ww groups of participants previously infected with SARS-CoV-2 (Fig. 4, Supplementary Table 6). On the contrary, no differences were found for IL-21 across groups (Supplementary Fig. 4, Supplementary Table 5).

### Safety of temporary MTX withdrawal

**Fig. 5:**
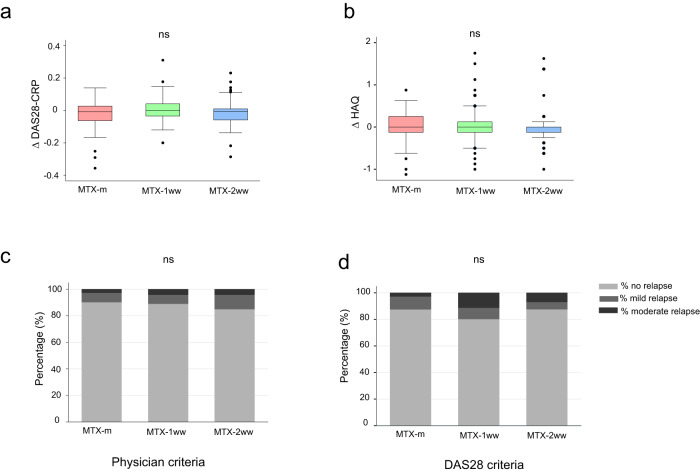
Safety evaluation of MTX withdrawal. **a** Increment of disease activity score in 28 joints using CRP between first and second visit (ΔDAS28-CRP) in MTX-m (*n* = 72), MTX-1ww (*n* = 71) and MTX-2ww (*n* = 73). **b** Increment of disability measured by the Health Assessment Questionnaire (ΔHAQ) in MTX-m (*n* = 72), MTX-1ww (*n* = 71) and MTX-2ww (*n* = 73). HAQ = 0 indicates no disability while a score of 3 indicates maximum disability. **c** Percentage of patients who relapsed during the study and severity of the relapse according to physician criteria. **d** Percentage of patients who relapsed during the study and severity of the relapse according to DAS28 criteria. Kruskal Wallis tests **a**, **b** or Fisher exact tests **c**, **d** were performed to assess statistical significance. The median of days between first and second visits was 77 (60–103) and the second visit took place a median of 29 days (28–35) after complete vaccination. Box plots represent the interquartile ranges, horizontal lines indicate the medians, and error bars extend to the upper and lower adjacent values.

A total of 32 (15%) patients showed a relapse of their RA/PsA according to DAS28 optimisation criteria (17 were classified as mild and 15 as moderate/severe) (Supplementary Table 7). An alternative method of flare classification was carried out according to physician-dependent criteria, which showed that 25 (12%) subjects had a relapse of their RA/PsA, classified as mild in seventeen patients and moderate in eight. In this case, delta disease activity in 28 joints calculated with C-reactive protein (DAS28-CRP) was 0.22 ± 1.03 in patients with relapse versus -0.17 ± 0.78 in those without relapse (*p* = 0.013). When taking into account the severity of the relapse, delta DAS28-CRP was 0.12 ± 0.86 and 0.43 ± 1.36 in patients with mild and moderate relapse, respectively. Remarkably, no significant differences in delta DAS28-CRP (*p* = 0.2) (Fig. 5a), delta health assessment questionnaire (HAQ) (*p* = 0.5) (Fig. 5b), number (*p* = 0.6) or severity (*p* = 0.9) of RA/PsA relapses were observed across groups when classifying by DAS28 or physician-dependent criteria (Fig. 5c, d). Higher baseline CRP was the only factor associated with relapse (OR 1.77, 95%CI [1.03–3.03]; *p* = 0.037) (Supplementary Table 8).

## Discussion

Our data show that a temporary MTX interruption after each vaccine dose improves both humoral and cellular adaptive immune responses in patients with RA and PsA, in particular in those without previous SARS-CoV2 infection. Remarkably, MTX withdrawal neither associates with a higher proportion of RA/PsA flares nor with the severity of the relapses. To the best of our knowledge, this is the largest randomised study exploring the effect of MTX withdrawal on both antibody and cellular responses upon SARS-CoV-2 vaccination and helps shed light on the controversial data regarding the impact of MTX on vaccination efficacy in immunocompromised patients.

Previous works addressing the study of the immune response after COVID-19 vaccination showed that patients with IMID had lower seroconversion rates after a single dose of vaccine^21,22^. Nevertheless, in most of them, humoral response improved after second and third doses, with antibody levels approaching those of healthy individuals^22,23^. According to our results, MTX discontinuation enhances not only total specific antibody levels, but also Nabs. These data are in accordance with previous studies describing a negative effect of DMARDs on adaptive immune response upon SARS-CoV-2 vaccination^24,25^. In fact, MTX was associated with a reduced antibody response in patients with IMID^26,27^, as well as with a lower CD8 + T cell activation capacity^27^. Taking this into account, MTX discontinuation, rather than dose adjustments, seems a plausible option to improve vaccine immune response^28^. Furthermore, temporary MTX interruption was associated with higher seroconversion rates after Sinovac-CoronaVac vaccine administration in a randomised study with 138 patients^17^ as well as with higher antibody titres in previous randomised trials^13,15^. In addition, our results agree with previous studies describing higher levels of Nabs in patients withdrawing MTX^14,16^.

Although no differences in levels of anti-S IgG response were identified between MTX-ww and MTX-m groups, our results show that temporary MTX withdrawal results in higher titres of total anti-RBD IgG antibodies, as well as higher neutralisation capacity. This observation suggests a higher quality of the immune response in those patients interrupting MTX.

Previous studies have shown that vaccination elicits IgA and IgM response in some individuals^29^, even in previously infected ones^30^. In our study, we found differences in IgA and IgM responses between MTX-ww and MTX-m groups although detection was limited. Probably, shorter sample collection time points from first and second dose would be necessary to draw firm conclusions about IgA and IgM titres and their kinetics following MTX discontinuation.

Moreover, we observed that the type of vaccine had an effect on both antibody and T cell responses, being mRNA vaccines more effective in this group of patients than vaccines using an adenovirus as vector, as it was formerly stated^31^. Consistent with previous data in the general population, vaccines with a 2-dose regimen elicited higher titres of antibodies than vaccines with a single-dose regimen such as Ad26.COV2.S^31^. Differences in number of doses across the studied groups could affect the immune response and, therefore, this fact should be taken into consideration when interpreting these results. Although we observed higher titres of anti-RBD antibodies and higher IFN-γ response in those suspending MTX, we cannot rule out that these differences were even wider if MTX-suspending groups would contain more two-vaccinated individuals (Supplementary Table 9). Furthermore, the age of our study population did not seem to have a high influence on antibody impairment mediated by MTX, as it was suggested elsewhere^32^.

Regarding cellular response, most participants had a detectable IFN-γ T cell response upon vaccination and a two-week MTX interruption improved this IFN-γ response in patients with RA or PsA, even in the case of individuals previously infected with SARS-CoV-2. These results agree with former published data showing that although T cell activation is not severely impaired in IMID patients, DMARDs may reduce T cell antiviral response^27,33^. Although both humoral and cellular responses seem to be affected by MTX, our data suggest that T cell activation is impeded by MTX to a greater extent. However, unlike what happened with humoral and IFN-γ response, no differences in IL-21 secretion were observed when MTX was stopped. IL-21 is a cytokine with a pleiotropic effect on the immune response and its secretion is restricted to natural killer T (NKT), T follicular helper (Tfh) and Th17 cells^34^. Our results agree with previous studies showing that MTX does not affect the Tfh subset, which is key for IL-21 secretion, after administering mRNA vaccines to patients with IMID^27^. Nonetheless, we cannot rule out that IL-21-producing cells may proliferate mainly upon a first exposure to the antigen, when all the repertoire of plasma and memory B cells is generated, so that successive exposures to the virus would not elicit a great IL-21 response. The fact that most patients showed low levels of IL-21 secretion may preclude from detecting differences among groups. Therefore, perhaps its study in a shorter time interval after vaccination would facilitate the detection of these IL-21-producing cells.

It is also important to evaluate the risk-benefit of temporary MTX suspension in order to avoid an increase in RA/PsA flares that could compromise the well-being of patients. In this study, safety of MTX withdrawal was assessed by means of DAS28-CRP and a HAQ, observing no significant differences in disease activity or HAQ among groups. Furthermore, most relapses in patients in the MTX-1ww and MTX-2ww groups were mild, suggesting that it is safe to temporarily withdraw MTX for up to two weeks after each vaccine dose, only if disease activity allows it.

This study has some limitations. First, this is a low-interventional randomised observational study, since time pressures of vaccination campaigns precluded us from registering it as a randomised clinical trial. It lacks a healthy control group, which could allow us to further deepen into the effect of MTX on immunogenicity of vaccination. In addition, our study population comprises patients on different treatment regimens, since many of them used MTX in combination with bDMARDs, other csDMARDs or tsDMARDs. However, non-MTX DMARDs remained unchanged in terms of dose and administration interval, which supports the role of MTX in the changes found on immunogenicity. In addition, different types of vaccines and IMID (RA and PsA) have been evaluated, and time from vaccine completion to final sample collection varied in some patients (interquartile range, IQR: 28–35) (Table 1), although these differences were balanced among MTX groups, making its interference unlikely. Furthermore, the fact that this is a randomised study with a large sample size (*n* = 216), in which data of humoral and cellular responses were corrected by a statistical multivariable analysis in order to rule out the effect of possible confounding variables, provides robustness to our results. In fact, multivariable analyses showed no influence of the type of IMID (RA or PsA) on post-vaccine immune responses. Conversely, we found that those participants who had COVID-19 before vaccination had generally a higher immune response after vaccine administration than those uninfected, even after receiving only one vaccine dose. Finally, DAS28 is a disease activity score commonly used for RA, but it has not been validated for PsA, since this score does not include joints that could be affected in PsA, enthesal affection and the degree of cutaneous involvement. Nevertheless, in order to homogenise disease activity evaluation in our study population, and, considering that PsA patients represented a small group in our study mainly suffering from polyarticular disease, we used DAS28 as it has been proposed elsewhere^35^.

Our results provide evidence that a two-week discontinuation of MTX after vaccination may be a safe strategy that improves immunogenicity of COVID-19 vaccines, especially in terms of Nabs and cellular response, in RA and PsA patients with adequate control of disease activity. However, in the case of difficult-to-treat patients that are not in remission, in which nocebo effects may occur, MTX withdrawal might not be necessary, in particular if they receive mRNA vaccines or have been previously infected with SARS-CoV-2. In addition, more studies are needed to draw firm conclusions regarding the effects of a temporary MTX withdrawal after other vaccination procedures, e.g. herpes zoster, since evidence of its good risk/benefit ratio has been reported with viral vaccines (influenza and COVID-19).

## Methods

### Study design and participants

This is a single-centre, randomised, prospective and investigator-blinded study carried out at a tertiary hospital (Hospital de La Princesa, Madrid, Spain) during the first COVID-19 vaccination campaign. However, the study could not be registered as a randomised clinical trial due to the time pressures of vaccination campaigns, which precluded a complete control of the intervention. To calculate sample size of the study population, we assumed an alpha error of 0.05, a power of 10%, a loss rate of 10% and an expected difference in antibody titres of 14%. Our estimated sample size per group was 68 patients (204 in total). Adult RA or PsA participants were enrolled between March and September 2021. The inclusion criteria were: to be diagnosed with RA or PsA according to ACR/EULAR 2010^36^ or CASPAR criteria^37^, respectively, ≥18 years of age, with stable MTX treatment for at least 6 weeks and willing to participate. Recruited participants were receiving MTX either as monotherapy or combined with other csDMARDs, bDMARDs or tsDMARDs. Exclusion criteria were pregnancy, having other IMID, Guillain-Barre syndrome, demyelinating disease, allergy to any component of the vaccine or having received attenuated or inactivated vaccines in the previous 4 or 2 weeks, respectively. Patients were randomly assigned to one of the three different groups of the study in a 1:1:1 ratio by an independent investigator, who was masked to patients’ evaluations. Randomisation was stratified by sex and age. Randomisation process was concealed from investigators in charge of enrolling and evaluating patients.

The type of COVID-19 vaccine administered to each patient was established by the local health authorities, following the vaccination protocol approved by the European Medicines Agency (EMA). For all those patients previously infected with SARS-CoV-2, our health authorities recommended a single dose of the BNT162b2, ChadOX-1-S and mRNA-1273 vaccines in the very early stages of the vaccination campaign (*n* = 19) (Supplementary Table 9). However, later on, these criteria were modified only for immunocompromised patients, including those with IMID, indicating the administration of two doses to this population even if they had suffered from COVID-19 prior to vaccination (*n* = 28). Two blood samples were collected for each participant: a baseline sample before vaccination (Supplementary Table 10) and the second one 4 weeks after completing the vaccination regimen, which consisted of two doses except for 38 individuals, either vaccinated with Ad26.COV2.S (single dose vaccine; *n* = 19), with BNT162b2 (*n* = 16), ChAdOx-1-S (*n* = 1) or mRNA-1273 (*n* = 2) (Supplementary Table 9). The interdose period between vaccines depended on the type of vaccine administered: 3, 4 or 10–12 weeks apart for BNT162b2, mRNA−1,273 and ChadOX-1-S vaccines, respectively. Routine laboratory testing and a thorough clinical evaluation of the patients, including DAS28-CRP and HAQ calculation, were also performed at these time points. Ethnicity was self-reported by study participants. Sex was assigned by researchers following external or internal examination of body characteristics.

Disease activity was defined according to DAS28 score: <2.6 remission; 2.6–3.2 low disease activity; >3.2–5.1 moderate disease activity; and >5.1 high disease activity^38^. Disease flares were defined by an increase in DAS28-CRP > 0.6. Flares were considered mild if there was evidence of an increase in DAS28-CRP > 0.6 and <1.2 and moderate/severe if increase in DAS28-CRP ≥ 1.2, provided that an intercurrent process (infection, surgery) was excluded^35^. Another gradation of flares was defined according to the physician criteria that included the intensity of joint symptoms (mild, moderate or severe) and the therapeutic adjustments required to control disease activity as follows: (a) mild, if resolved with non-steroidal anti-inflammatory drugs (NSAIDs) or a short cycle of corticosteroids; (b) moderate, if mild MTX dose adjustment was required; and (c) severe, if therapeutic scalation was needed (addition or switch to a biologic or targeted directed DMARD).

### Ethics statement

This study was approved by the local Research Ethics Committee (register 4590/2021) and it was carried out following the ethical principles established in the Declaration of Helsinki. All recruited patients were informed about the study and signed the informed consent before their inclusion in the study.

### Serum antibody assessment

Blood samples were collected by peripheral venipuncture and serum was obtained by centrifugation at 800 g for 10 min, aliquoted and stored at -20 °C until use. Assessment of antibody levels against Mpro, NP, S and RBD in serum was performed using a SARS-CoV-2 multi-antigen cytometric bead array (Immunostep), following manufacturer’s instructions. Median fluorescence intensity (MFI) was measured with a FACS Lyric Cytometer (Becton Dickinson) and analysed with FlowJo V10 (Becton Dickinson). Samples were randomly included in each plate and a pool of sera positive for specific antibodies was used as inter-plate control. Cut-off for positivity was determined as (µ+ 3 SD) × 1.2, where µ is the average of MFI of negative control samples.

Nabs were detected by ELISA using the SARS-CoV-2 Neutralisa kit (Euroimmun). Briefly, sera were diluted in buffer containing soluble biotinylated ACE2 and 100 µl of samples or controls were added to each plate well, each of them coated with a recombinant RBD of SARS-CoV-2. Plate was incubated for 1 h at 37 °C. Wells were washed 3 times with 300 µl of washing buffer. Then 100 µl of enzyme conjugate was added and incubated for 30 min. Plates were washed again and incubated in the dark with 100 µl/well of substrate solution for 15 min at room temperature. Finally, 100 µl of stop solution were added and plates were read at 450 nm (reference wavelength of 620 nm), using a Glomax® Discover Microplate Reader (Promega). Manufacturer’s recommended cut-off for positivity (25% of neutralisation) was employed.

### PBMC isolation and ELISPOT assays

Peripheral blood mononuclear cells (PBMCs) were harvested from whole blood via Ficoll-Paque (Pan-Biotech) and subsequently cryopreserved in foetal bovine serum (FBS; HyClone TM) with 10% DMSO (Inilab). We decided to assess IFN-y and IL-21 secretion in PBMCs from patients as a readout of T cell response. IFN-y, which is mainly produced by natural killer cells, CD4 Th1 cells and CD8 T cells, is a well-known cytokine that promotes activation of multiple antiviral mechanisms^39^. On the other hand, IL-21 secretion by Th17 and Tfh subsets is involved in CD8 T cell activation and proliferation^18^, as well as in B cell differentiation and antibody production, helping germinal centre reactions and antibody class-switching^40,41^. IFN-γ/IL-21 ELISPOT assays were performed following manufacturer’s instructions (Immunospot, Cellular Technology Ltd.). In brief, plates were incubated overnight at 4 °C with anti-IFN-γ and anti-IL-21 capture antibodies. PBMCs were thawed, centrifuged 500 g 5 min and resuspended in RPMI 1640 + GlutaMax^TM^ (GIBCO) with 10% FBS and 1% penicillin-streptomycin (Biowest). Then, cells were incubated for 2–3 h at a concentration of 1-2 × 10^6^/mL at 37 °C and 5% CO_2_ atmosphere. Afterwards, PBMCs were harvested, centrifuged, counted in an automated cell counter (Nihon Koden) and plated at a final density of 2 × 10^5^ cells/well. Cellular responses were assessed by using three different SARS-CoV-2 peptide pools spanning the S1, S2 and RBD domains of the S protein (1.25 µg/mL) (PepMix TM, JPT peptide Technologies). As a negative control, a pool of peptides spanning actin was used. To help cell stimulation, 1 µg/mL of CD28 antibody, anti-human, pure-functional grade, clone 15E8 (Miltenyi Biotec) was added to culture medium. As a positive control, 1 × 10^5^ cells/well were plated in the presence of staphylococcal enterotoxin B (SEB; Sigma-Aldrich) at 100 ng/mL. Plates were incubated 16 h at 37 °C and the spots were revealed following manufacturer’s protocol. ELISPOT plates were analysed using an AID classic ELISPOT reader (AID Autoimmun Diagnostika Gmbh). Samples were randomly included in each plate and a sample from a vaccinated healthy donor was used as a positive inter-plate control.

Samples with a high response upon actin stimulation ( > 100 SFU/10^6^) were excluded from subsequent analyses. ELISPOT positivity cut-off was determined as the mean + 2 SD of the spot forming units (SFU) of all samples tested upon actin stimulation.

### Statistical analysis

Statistical analyses were performed using Stata 14 for Windows (Stata Corp LP, College Station, Tex, USA). Quantitative variables were represented as median and IQR, and the ANOVA test for normally distributed variables or Kruskal Wallis test for variables following a non-normal distribution were used to assess significant differences. Qualitative variables were described as counts and proportions, and chi-square or Fisher exact test were used for comparisons.

Considering that several variables like sex, age, previous SARS-CoV-2 infection, MTX dose, ethnicity, smoking habit, DAS28-CRP, lymphocyte count, type of vaccine, inter-plate variability and comorbidities, among others, could influence response to vaccination, different multivariable analyses were fitted, including as independent variables those with a p-value < 0.15 in the bivariate analyses with the respective dependent variables. For humoral responses, we used as dependent variable the increase in antibody levels between second and first visits, expressed as arbitrary units/mL (AU/mL). In the case of cellular response, since most baseline measurements were negligible, even in many patients previously infected with SARS-CoV-2, the post-vaccination results were used as dependent variables. Cellular responses (SFU/10^6^) were transformed to logarithm or square root in the case they did not follow a normal distribution.

To determine which variables could explain variability in humoral and cellular response to SARS-CoV-2 vaccination, we performed a multivariable linear regression model using generalised linear models (GLM) for each dependent variable by using the Stata *glm* command. Analysis was first modelled by adding all the variables with a *p*-value lower than 0.15 in the bivariable analysis. The final model was reached by means of a backward stepwise removal of variables with *p*-value > 0.15, except when the removal of a variable worsened the goodness of the model, defined as an increase of Akaike information criteria. Once each final multivariable analysis was obtained, the variable MTX withdrawal (no withdrawal, 1-ww or 2-ww) was forced in the model. To achieve a more accurate graphical representation, the figures shown in this work were depicted using the new adjusted variables generated with the postestimation command of Stata *predict*, after the final multivariable models (see Supplementary Tables 1 to 4) were run. Statistical significance across groups for these new adjusted variables was checked with the Cuzick test, an extension of the Wilcoxon rank-sum test for trend across ordered groups (*nptrend* command of Stata). In addition, to further assess the relevance of MTX withdrawal upon vaccine administration, the outcome variables for humoral and cellular response were categorically classified in three subgroups: poor responders, if no response or a response below 25^th^ percentile of positive responders was achieved; moderate responders for those between 25^th^ and 50^th^ percentiles; and good responders for those above the 50^th^ percentile.

To evaluate potential factors associated with RA/PsA relapses, similar multivariable analyses were carried out, including age, sex, glucocorticoid therapy, dose of MTX, rheumatoid factor, presence of nodules, asthma, treatment with other DMARDs and the group of MTX withdrawal as independent variables.

Graphs were depicted with GraphPad Prism 8 for Windows and RStudio 2021.09.0.

### Supplementary information

Supplementary material

## Data Availability

All data relevant to the study are included in the article. Additional data are available upon reasonable request.
