# Supplementary material for: Beneficial effect of temporary methotrexate interruption on B and T cell responses upon SARS-CoV-2 vaccination in patients with rheumatoid arthritis or psoriatic arthritis
Source: NPJ Vaccines. 2024 Jan 30;9:21. doi: 10.1038/s41541-024-00805-3 (PMC10827732; doi:10.1038/s41541-024-00805-3)
Supplement: Supplementary file 1 — Supplementary material [file 41541_2024_805_MOESM1_ESM.pdf]

SUPPLEMENTARY MATERIAL

Beneficial effect of temporary methotrexate interruption on B and T cell responses upon SARS-CoV-2 vaccination in patients with rheumatoid arthritis or psoriatic arthritis

Pedro Martínez-Fleta[1†], Esther F. Vicente-Rabaneda[2†], Ana Triguero-Martínez[2*], Emilia Roy-Vallejo[3*], Miren Uriarte-Ecenarro[2], Francisco Gutiérrez-Rodríguez[2], Patricia Quiroga-Colina[2], Ana Romero-Robles[2], Nuria Montes[2], Noelia García-Castañeda[2], Gina P. Mejía-Abril[4], Jesús A. García-Vadillo[2], Irene Llorente-Cubas[2], José R. Villagrasa[5], José M. Serra López-Matencio[6], Julio Ancochea[7,8,9], Ana Urzainqui[1], Laura Esparcia-Pinedo[1], Arantzazu Alfranca[1,8,10], Hortensia de la Fuente[1,10], Rosario García-Vicuña[2,8], Francisco Sánchez-Madrid[1,8,10], Isidoro González-Álvaro[2Ω], Santos Castañeda[2,9Ω].

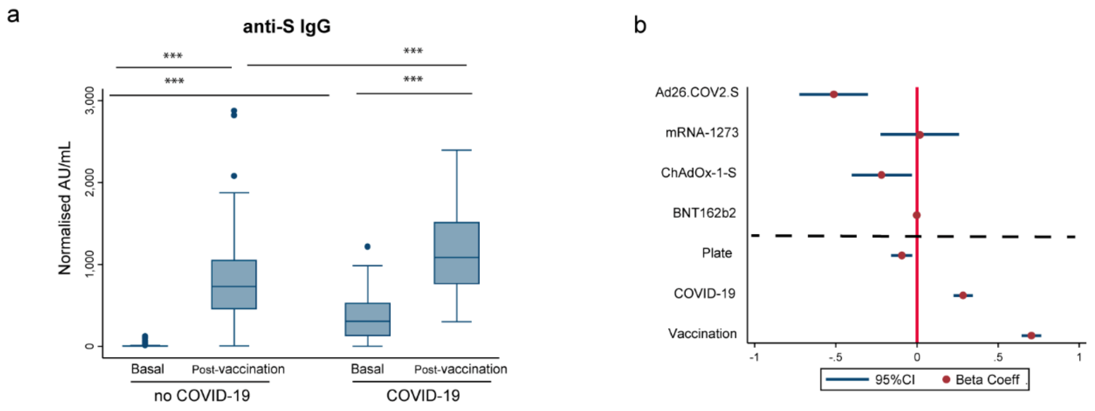

Supplementary figure 1. **Effect of previous COVID-19 and type of vaccine on antibody response. a)** Antibody response (anti-S IgG) expressed as normalised AU/mL before vaccination and 1 month after complete vaccination in previously uninfected (no COVID-19) (n=169) and previously infected (COVID-19) (n=47) individuals. Statistical significance was assessed by means of a generalised linear model. ***$p<0.001$. **b)** Standardised Beta Coefficients and 95% CI of type of vaccine, plate number, previous COVID-19 and vaccination of the generalised linear model used for anti-S IgG. Box plots represent the interquartile ranges, horizontal lines indicate the medians, and error bars extend to the upper and lower adjacent values.

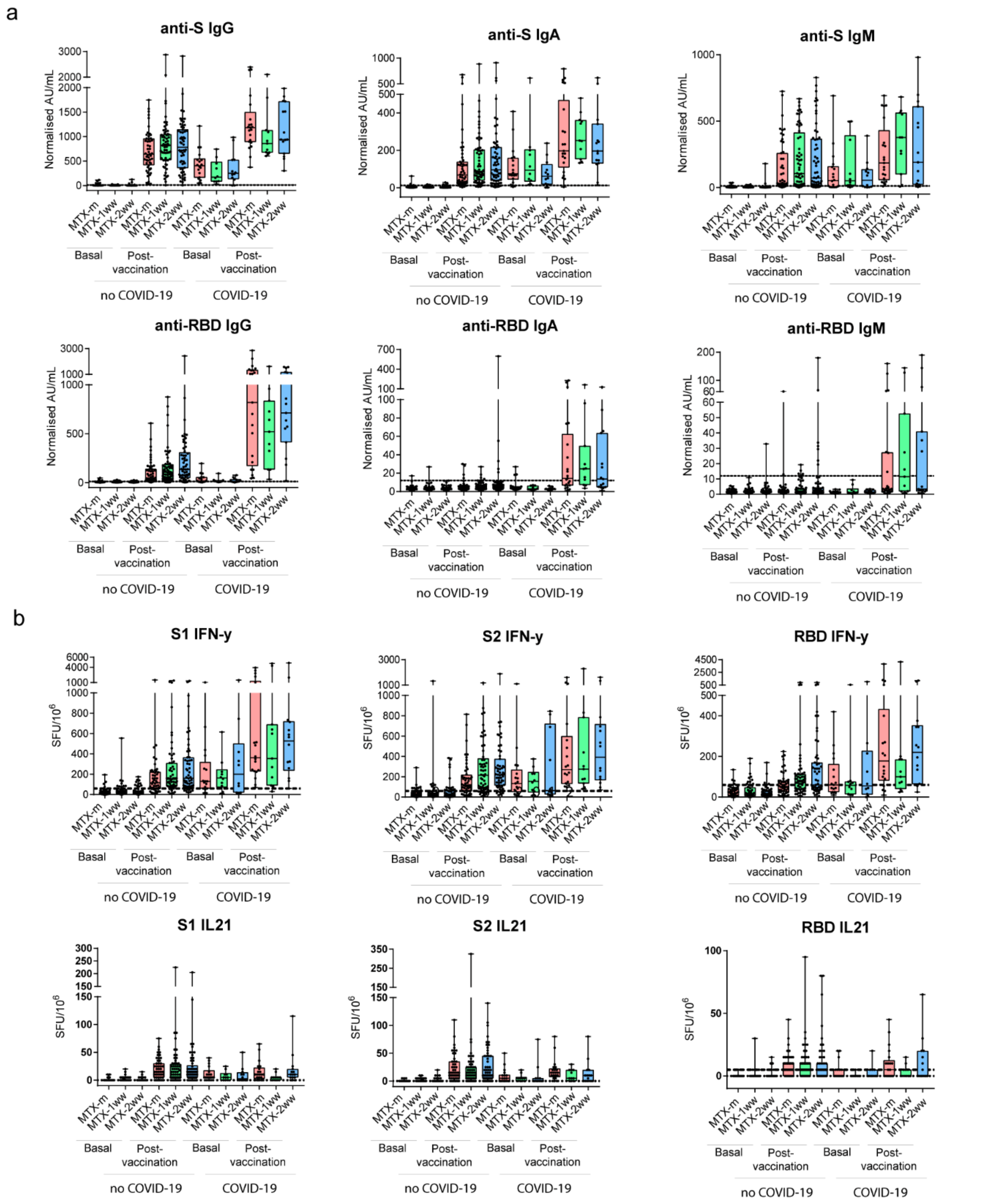

Supplementary figure 2. **Baseline and post-vaccination antibody and cellular responses**. **a)** Specific antibody response (IgG, IgA and IgM) against S and RBD expressed as AU/mL before vaccination and 1 month after complete vaccination in MTX-m (n=72), MTX-1ww (n=71) and MTX-2ww (n=73) groups, separated in previously uninfected (no COVID-19) and previously infected (COVID-19) individuals. **b)** IFN-γ and IL-21 responses

in MTX-m (n=66), MTX-1ww (n=68) and MTX-2ww (n=69) groups upon stimulation with S1, S2 and RBD peptide pools, before vaccination and 1 month after complete vaccination in previously uninfected (no COVID-19) and previously infected (COVID-19) individuals. Positivity threshold is shown as a dotted line. Box plots represent the interquartile ranges, horizontal lines indicate the medians, and error bars extend to the minimum and maximum observed values.

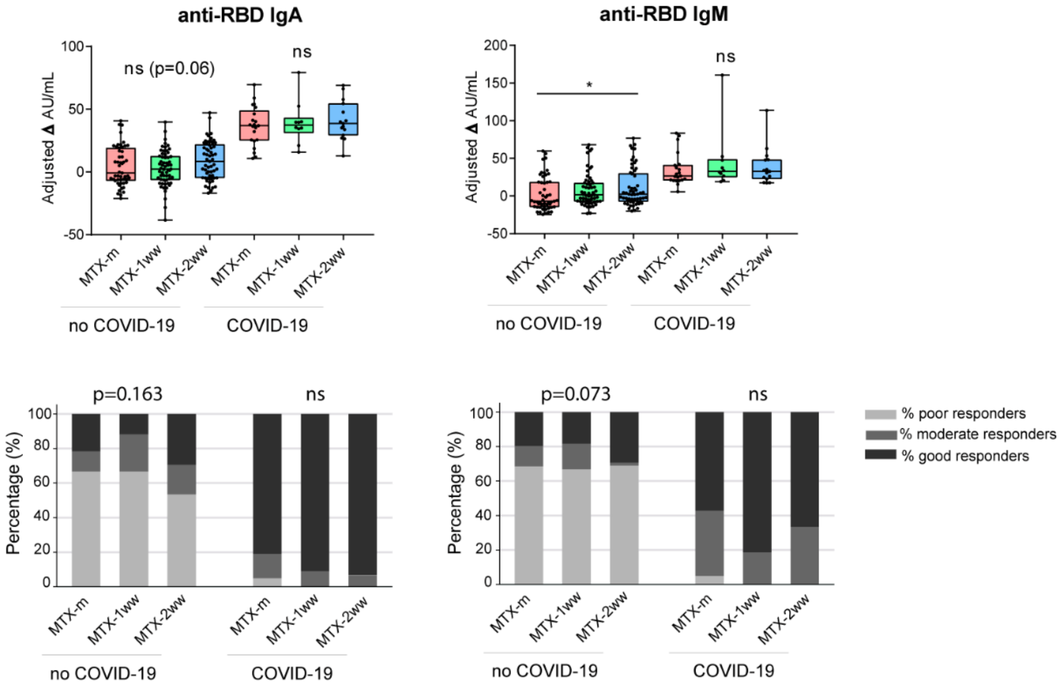

Supplementary figure 3. **Anti-RBD IgA and IgM antibody response**. Specific antibody response (IgA and IgM) against RBD before vaccination and 1 month after complete vaccination in MTX-m (n=72), MTX-1ww (n=71) and MTX-2ww (n=73) groups separated in previously uninfected (no COVID-19) and previously infected (COVID-19) individuals. Results are represented as increment (Δ) of AU/mL between first and second visit, after adjusting for confounding variables by means of glm (upper panels). Cuzick test was used to assess statistical significance. *p<0.05 (up). Percentage of poor (below 25th percentile response), moderate (25th to 50th percentile) and good responders (greater than 50th percentile) to vaccination across groups (lower panels). Fisher exact test was used to assess statistical significance. Box plots represent the interquartile ranges, horizontal lines indicate the medians, and error bars extend to the minimum and maximum observed values.

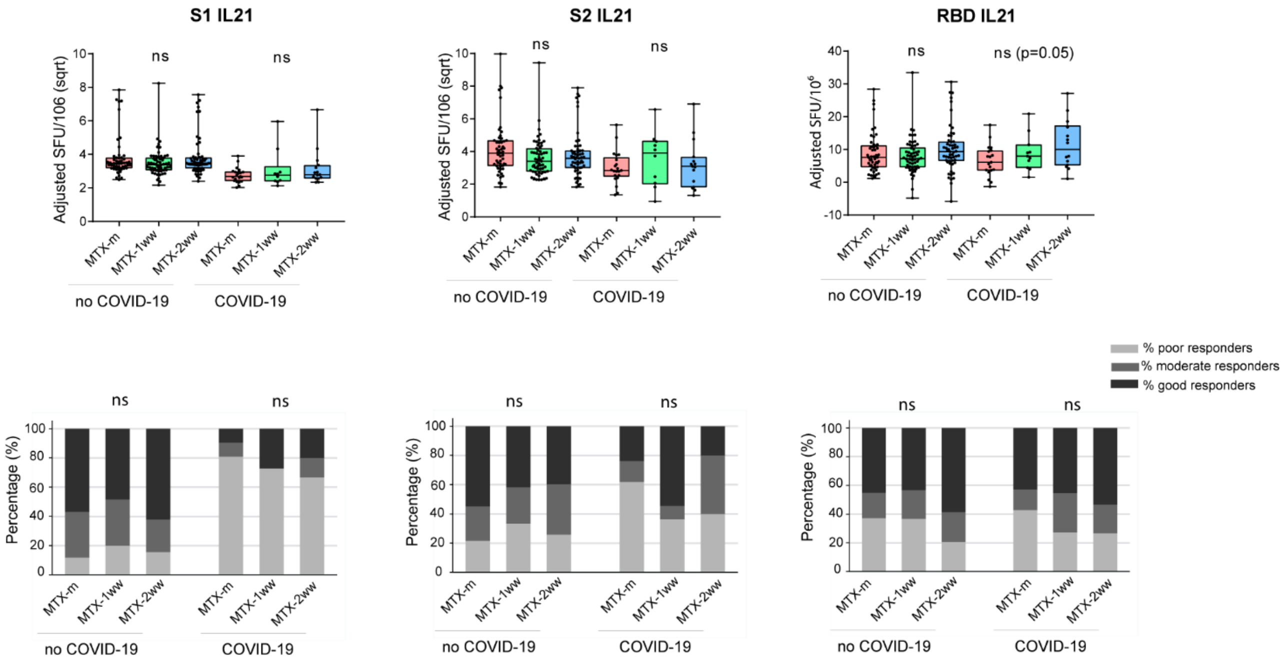

Supplementary figure 4. **Cellular response of IL-21 after vaccine administration.** IL-21 response 1 month after complete vaccination in MTX-m (n=72), MTX-1ww (n=71) and MTX-2ww (n=73) in previously uninfected (no COVID-19) and previously infected (COVID-19) individuals, after adjusting for confounding variables by means of glm. PBMCs were stimulated with pools of peptides spanning S1, S2 and RBD. Cuzick test was used to assess statistical significance. **p<0.01 (upper panels). Percentage of poor (below 25th percentile response), moderate (25th to 50th percentile) and good responders (greater than 50th percentile) to vaccination across groups. Fisher exact test was used to test for statistical significance (lower panels). Box plots represent the interquartile ranges, horizontal lines indicate the medians, and error bars extend to the minimum and maximum observed values.

Supplementary Table 1. Multivariable analyses of anti-S antibodies.

**Anti-S IgG (ΔAU/mL)**	**Coef.**	**Std. Err.**	**z**	***p***	**95% CI**	
previous COVID-19	206.7009	88.90603	2.32	0.02	32.44824	380.9535
Plate Nº						
3	243.2055	199.3886	1.22	0.223	-147.5889	633.9999
4	933.0641	203.8169	4.58	0.000	533.5904	1332.538
5	904.2558	199.4501	4.53	0.000	513.3408	1295.171
6	861.1297	202.6821	4.25	0.000	463.8801	1258.379
7	52.6195	198.6888	0.26	0.791	-336.8035	442.0425
8	488.1227	198.9719	2.45	0.014	98.14504	878.1004
9	608.3485	202.7308	3	0.003	211.0034	1005.694
10	597.0746	195.2251	3.06	0.002	214.4404	979.7089
11	259.5483	200.1615	1.3	0.195	-132.761	651.8575
12	534.2992	199.7408	2.67	0.007	142.8144	925.7841
13	318.8855	213.5665	1.49	0.135	-99.69721	737.4682
Smoker						
Active	-75.39689	112.4485	-0.67	0.503	-295.792	144.9982
Former	-122.0366	83.59287	-1.46	0.144	-285.8756	41.80242
Vaccine type (BNT162b2)						
ChAdOX-1-S	-216.2356	115.0876	-1.88	0.06	-441.8032	9.331936
mRNA-1273	119.612	148.9197	0.8	0.422	-172.2653	411.4892
Ad26.COV2.S	-640.97	129.3618	-4.95	0.000	-894.5145	-387.4256
MTX withdrawal (MTX-m)						
MTX-1ww	131.4177	89.62735	1.47	0.143	-44.24866	307.0841
MTX-2ww	60.61229	87.25907	0.69	0.487	-110.4123	231.6369
Constant	350.9996	167.1249	2.1	0.036	23.44091	678.5584

**Anti-S IgA (ΔAU/mL)**	**Coef.**	**Std. Err.**	**z**	***p***	**95% CI**	
Sex						
Female	46.772	26.75806	1.75	0.08	-5.672843	99.21684
previous COVID-19	38.72348	25.72359	1.51	0.132	-11.69384	89.14079
Plate Nº						
3	152.5251	57.11289	2.67	0.008	40.58584	264.4643
4	61.43078	58.58087	1.05	0.294	-53.38562	176.2472
5	11.64778	57.83962	0.2	0.84	-101.7158	125.0114
6	-68.39194	56.96379	-1.2	0.23	-180.0389	43.25504
7	-15.14191	57.0293	-0.27	0.791	-126.9173	96.63347
8	-3.222557	56.14896	-0.06	0.954	-113.2725	106.8274
9	39.42758	57.28044	0.69	0.491	-72.84003	151.6952
10	-9.472303	56.0767	-0.17	0.866	-119.3806	100.436
11	-35.09582	58.08158	-0.6	0.546	-148.9336	78.74198
12	-25.48335	57.41247	-0.44	0.657	-138.0097	87.04301
13	-6.580533	62.19663	-0.11	0.916	-128.4837	115.3226
Vaccine type (BNT162b2)						
ChAdOX-1-S	-0.4259537	33.58315	-0.01	0.99	-66.24771	65.3958
mRNA-1273	-3.141255	43.03636	-0.07	0.942	-87.49097	81.20846
Ad26.COV2.S	-42.32175	37.34626	-1.13	0.257	-115.5191	30.87557
MTX withdrawal (MTX-m)						
MTX-1ww	18.16727	25.56319	0.71	0.477	-31.93566	68.27021
MTX-2ww	30.11979	25.19449	1.2	0.232	-19.26051	79.5001
Constant	59.26008	53.45081	1.11	0.268	-45.50158	164.0218

**Anti-S IgM (ΔAU/mL)**	**Coef.**	**Std. Err.**	**z**	***p***	**95% CI**	
Sex						
Female	158.6489	50.107	3.17	0.002	60.441	256.8568
Plate №						
3	174.4194	106.6078	1.64	0.102	-34.52797	383.3668
4	267.8707	109.2441	2.45	0.014	53.75618	481.9852
5	169.2992	107.0079	1.58	0.114	-40.43253	379.0309
6	15.00948	106.2555	0.14	0.888	-193.2475	223.2664
7	147.3824	107.3953	1.37	0.17	-63.10848	357.8732
8	106.4791	106.4738	1	0.317	-102.2057	315.164
9	131.9922	106.7693	1.24	0.216	-77.27185	341.2562
10	12.22095	104.3936	0.12	0.907	-192.3868	216.8287
11	138.0003	107.6852	1.28	0.2	-73.05882	349.0594
12	71.83067	106.3657	0.68	0.499	-136.6423	280.3036
13	62.20404	115.6607	0.54	0.591	-164.4868	288.8949
Ethnicity (White)						
Latin American	109.6398	58.36212	1.88	0.06	-4.747878	224.0274
Black	-135.6204	210.1998	-0.65	0.519	-547.6044	276.3635
North African	46.24971	287.7415	0.16	0.872	-517.7132	610.2126
Vaccine type (BNT162b2)						
ChAdOX-1-S	26.6599	62.86587	0.42	0.672	-96.55494	149.8747
mRNA-1273	17.84131	80.57908	0.22	0.825	-140.0908	175.7734
Ad26.COV2.S	-127.5899	69.54966	-1.83	0.067	-263.9047	8.72498
MTX withdrawal (MTX-m)						
MTX-1ww	54.8264	47.39785	1.16	0.247	-38.07168	147.7245
MTX-2ww	60.91978	47.50423	1.28	0.2	-32.18679	154.0264
Constant	-65.8154	99.44692	-0.66	0.508	-260.7278	129.097

Coef, β-coefficient; std.err, standard error; CI, confidence interval. Final generalised linear models after backward stepwise removal of variables with no influence in the model. $p<0.05$ was considered statistically significant.

Supplementary Table 2. Multivariable analyses of anti-RBD antibodies.

Anti-RBD IgG (ΔAU/mL)	Coef.	Std. Err.	z	*p*	95% CI	
previous COVID-19	515.0908	47.00928	10.96	0.000	422.9543	607.2273
Plate Nº						
3	242.5926	105.5362	2.3	0.022	35.7455	449.4397
4	333.552	106.6949	3.13	0.002	124.4339	542.6701
5	203.2054	104.292	1.95	0.051	-1.203112	407.6139
6	324.7737	106.5215	3.05	0.002	115.9954	533.5519
7	138.9278	105.3166	1.32	0.187	-67.48891	345.3445
8	320.7784	106.7416	3.01	0.003	111.5688	529.9881
9	253.1938	106.3302	2.38	0.017	44.79041	461.5972
10	174.2501	102.1882	1.71	0.088	-26.03508	374.5353
11	214.3946	104.7732	2.05	0.041	9.042949	419.7462
12	70.36705	105.294	0.67	0.504	-136.0054	276.7395
13	139.1371	111.4832	1.25	0.212	-79.36589	357.6402
Ethnicity (White)						
Latin American	100.9895	59.81983	1.69	0.091	-16.25518	218.2342
Black	-272.5701	207.4817	-1.31	0.189	-679.2268	134.0867
North African	-79.11674	278.9901	-0.28	0.777	-625.9274	467.6939
Smoker						
Active	-86.60973	60.63795	-1.43	0.153	-205.4579	32.23846
Former	1.097412	45.2148	0.02	0.981	-87.52197	89.7168
Days after vaccination	-4.165666	1.479299	-2.82	0.005	-7.065039	-1.266292
Vaccine type (BNT162b2)						
ChAdOX-1-S	-95.66975	61.11098	-1.57	0.117	-215.4451	24.10556
mRNA-1273	75.05244	77.98097	0.96	0.336	-77.78745	227.8923
Ad26.COV2.S	-232.2212	67.66317	-3.43	0.001	-364.8385	-99.60379
MTX withdrawal (MTX-m)						
MTX-1ww	1.170014	47.33345	0.02	0.98	-91.60183	93.94186
MTX-2ww	54.40619	46.22384	1.18	0.239	-36.19087	145.0032
Constant	75.14135	99.20612	0.76	0.449	-119.2991	269.5818

**Anti-RBD IgA (ΔAU/mL)**	**Coef.**	**Std. Err.**	**z**	***p***	**95% CI**	
previous COVID-19	32.09866	8.19463	3.92	0.000	16.03748	48.15984
Plate Nº						
3	39.94511	17.77767	2.25	0.025	5.101508	74.7887
4	27.35974	18.10496	1.51	0.131	-8.125336	62.84481
5	1.590035	17.70332	0.09	0.928	-33.10783	36.2879
6	6.888333	17.8634	0.39	0.7	-28.12329	41.89995
7	24.74985	17.90106	1.38	0.167	-10.33558	59.83528
8	2.435645	17.85493	0.14	0.891	-32.55937	37.43066
9	1.279208	17.53329	0.07	0.942	-33.0854	35.64382
10	-2.102771	17.25015	-0.12	0.903	-35.91245	31.7069
11	12.69887	17.87143	0.71	0.477	-22.32848	47.72622
12	15.64757	17.82557	0.88	0.38	-19.28991	50.58504
13	0.2215809	19.04872	0.01	0.991	-37.11323	37.55639
Ethnicity (White)						
Latin American	16.63499	9.878641	1.68	0.092	-2.726791	35.99677
Black	9.073503	35.42104	0.26	0.798	-60.35047	78.49747
North African	-27.36256	47.57184	-0.58	0.565	-120.6017	65.87653
DAS28	6.109676	3.56487	1.71	0.087	-0.8773399	13.09669
Days after vaccination	-0.5069538	0.2532427	-2	0.045	-1.0033	-0.0106072
Vaccine type (BNT162b2)						
ChAdOX-1-S	-18.83947	10.44468	-1.8	0.071	-39.31068	1.631731
mRNA-1273	15.26666	13.31639	1.15	0.252	-10.83299	41.3663
Ad26.COV2.S	-11.03774	11.73827	-0.94	0.347	-34.04432	11.96885
MTX withdrawal (MTX-m)						
MTX-1ww	-0.3752802	7.987597	-0.05	0.963	-16.03068	15.28012
MTX-2ww	0.6584617	7.906155	0.08	0.934	-14.83732	16.15424
Constant	-2.375589	18.59671	-0.13	0.898	-38.82447	34.0733

Anti-RBD IgM (ΔAU/mL)	Coef.	Std. Err.	z	*p*	95% CI	
previous COVID-19	30.89973	10.73209	2.88	0.004	9.865216	51.93424
Plate Nº						
3	31.78363	23.18529	1.37	0.17	-13.6587	77.22595
4	66.46982	23.8363	2.79	0.005	19.75152	113.1881
5	-5.211431	23.3799	-0.22	0.824	-51.03519	40.61232
6	-0.4850596	23.45013	-0.02	0.983	-46.44648	45.47636
7	2.575933	23.34621	0.11	0.912	-43.18181	48.33367
8	-6.281554	23.11354	-0.27	0.786	-51.58326	39.02015
9	-2.277385	23.20942	-0.1	0.922	-47.76702	43.21225
10	-6.524068	22.8331	-0.29	0.775	-51.27613	38.22799
11	3.176124	23.61658	0.13	0.893	-43.11152	49.46376
12	1.785405	23.36198	0.08	0.939	-44.00323	47.57404
13	-1.987399	25.2217	-0.08	0.937	-51.42102	47.44622
DAS28	9.80942	4.695773	2.09	0.037	0.6058751	19.01297
Vaccine type (BNT162b2)						
ChAdOX-1-S	-11.97496	13.58947	-0.88	0.378	-38.60984	14.65991
mRNA-1273	45.30884	17.52557	2.59	0.01	10.95935	79.65832
Ad26.COV2.S	-12.12846	15.49653	-0.78	0.434	-42.50109	18.24417
MTX withdrawal (MTX-m)						
MTX-1ww	9.454491	10.48608	0.9	0.367	-11.09785	30.00684
MTX-2ww	6.734565	10.28	0.66	0.512	-13.41386	26.88299
Constant	-27.78577	22.80291	-1.22	0.223	-72.47865	16.9071

Coef, β-coefficient; std.err, standard error; CI, confidence interval. Final generalised linear models after backward stepwise removal of variables with no influence in the model. $p<0.05$ was considered statistically significant.

Supplementary table 3. Antibody response and percentage of responders after vaccination.

	MTX-m (n=72)		MTX-1ww (n=71)		MTX-2ww (n=73)	
	No COVID-19 (n=51)	COVID-19 (n=21)	No COVID-19 (n=60)	COVID-19 (n=11)	No COVID-19 (n=58)	COVID-19 (n=15)
**anti-S IgG (ΔAU/mL)**	809.9 (472.2-917)	970 (817.2-1202.6)	955 (535-1079.5)	1055.2 (350.8-1223.4)	868.4 (532.8-1008.7)	1104 (802.5-1215.4)
**Responders**	51 (100)	21 (100)	59 (98.3)	11 (100)	57 (98.3)	15 (100)
**Poor**	17 (33.3)	1 (4.8)	16 (26.7)	3 (27.3)	20 (34.5)	1 (6.7)
**Moderate**	19 (37.3)	8 (38.1)	12 (20)	1 (9.1)	9 (15.5)	4 (26.7)
**Good**	15 (29.4)	12 (57.1)	32 (53.3)	7 (63.6)	29 (50)	10 (66.7)
**anti-S IgA (ΔAU/mL)**	96.6 (56-117.2)	129.6 (109.6-156)	115.8 (73.3-135.6)	137.4 (106.7-162.5)	128.8 (110.7-147.8)	165.4 (139.8-186.1)
**Responders**	45 (88.2)	20 (95.2)	55 (91.7)	11 (100)	54 (93.1)	14 (93.3)
**Poor**	19 (37.2)	5 (23.8)	17 (28.3)	2 (18.2)	9 (15.5)	2 (13.3)
**Moderate**	20 (39.2)	5 (23.8)	21 (35)	1 (9.1)	8 (13.8)	1 (6.7)
**Good**	12 (23.5)	11 (52.4)	22 (36.7)	8 (72.7)	41 (70.7)	12 (80)
**anti-S IgM (ΔAU/mL)**	164.7 (92.8-240.2)	164.7 (105.1-340.5)	227.8 (159.1-318.2)	219.5 (158.3-267.7)	260.2 (166-309.6)	285.7 (183.8-349.7)
**Responders**	37 (72.5)	21 (100)	50 (83.3)	10 (90.9)	44 (75.9)	14 (93.3)
**Poor**	24 (47.1)	9 (42.9)	12 (20)	2 (18.2)	7 (12.1)	3 (20)
**Moderate**	10 (19.6)	3 (14.3)	18 (30)	4 (36.4)	14 (24.1)	2 (13.3)
**Good**	17 (33.3)	9 (42.9)	30 (50)	5 (45.5)	37 (63.8)	10 (66.7)
**anti-RBD IgG (ΔAU/mL)**	118.7 (42.3-202.2)	624.9 (536.7-713.5)	110.6 (15.3-201.9)	553.5 (495.1-784.1)	185.4 (88.9-292.1)	731.2 (624.1-837.2)
**Responders**	39 (76.5)	21 (100)	53 (88.3)	11 (100)	50 (86.2)	15 (100)
**Poor**	28 (54.9)	0 (0)	32 (53.3)	0 (0)	20 (34.5)	0 (0)
**Moderate**	14 (27.5)	0 (0)	15 (25)	0 (0)	15 (25.9)	0 (0)
**Good**	9 (17.6)	21 (100)	13 (21.7)	11 (100)	23 (39.7)	15 (100)
**anti-RBD IgA (ΔAU/mL)**	-0.8 (-7-19)	37 (25.5-46.3)	2.4 (-6.3-12.7)	37.5 (34.4-40)	8.4 (-4.6-21.3)	38.6 (29.3-54.5)
**Responders**	4 (7.8)	10 (47.6)	4 (6.7)	7 (63.6)	6 (10.3)	7 (46.7)
**Poor**	34 (66.7)	1 (4.8)	40 (66.7)	0 (0)	31 (53.4)	0 (0)
**Moderate**	6 (11.8)	3 (14.3)	13 (21.7)	1 (9.1)	10 (17.2)	1 (6.7)
**Good**	11 (21.6)	17 (81)	7 (11.7)	10 (90.9)	17 (29.3)	14 (93.3)
**anti-RBD IgM (ΔAU/mL)**	-7.1 (-14.5-18.4)	26.6 (21-40.7)	1.7 (-7.1-17.4)	32.7 (26.5-47.5)	2.4 (-7.1-29.4)	32.6 (22.5-48.3)
**Responders**	3 (5.9)	7 (33.3)	5 (8.3)	5 (45.5)	8 (13.8)	7 (46.7)
**Poor**	35 (68.6)	1 (4.8)	40 (66.7)	0 (0)	40 (69)	0 (0)
**Moderate**	6 (11.8)	8 (38.1)	9 (15)	2 (18.2)	1 (1.7)	5 (33.3)
**Good**	10 (19.6)	12 (57.1)	11 (18.3)	9 (81.8)	17 (29.3)	10 (66.7)
**% Neutralisation**	75.2 (54.2-95.3)	99.1 (98.5-99.2)	94.8 (80.7-98.2)	99.2 (98.7-99.3)	96.7 (78.5-98.9)	99.2 (99.1-99.2)
**Positivity for neutralisation**	46 (90.2)	21 (100)	55 (91.7)	11 (100)	51 (87.9)	15 (100)

Estimated ΔAU/mL using the final multivariable models are shown. All categorical variables are expressed as absolute count (percentage) and quantitative variables as median (IQR).

Supplementary table 4. Multivariable analyses of IFN-γ response.

Log S1 IFN-γ (SFU/10^6)	Coef.	Std. Err.	z	*p*	95% CI	
previous COVID-19	1.179352	0.1801234	6.55	0.000	0.8263161	1.532387
Peptide batch						
2	0.605828	0.2369583	2.56	0.011	0.1413984	1.070258
3	0.5980427	0.2477892	2.41	0.016	0.1123848	1.083701
4	1.115577	0.2583248	4.32	0.000	0.6092701	1.621885
5	0.8558721	0.2482655	3.45	0.001	0.3692806	1.342464
6	0.6384659	0.2813446	2.27	0.023	0.0870406	1.189891
Ethnicity (White)						
Latin American	0.4117749	0.2305821	1.79	0.074	-0.0401576	0.8637075
Black	-1.142626	0.9935866	-1.15	0.25	-3.09002	0.8047679
North African	1.231377	0.9933158	1.24	0.215	-0.7154857	3.178241
DAS28	-0.1810397	0.0818656	-2.21	0.027	-0.3414933	-0.020586
Lymphocyte count	-0.0002105	0.0001015	-2.07	0.038	-0.0004095	-0.0000114
Vaccine type (BNT162b2)						
ChAdOX-1-S	-0.3164765	0.2359046	-1.34	0.18	-0.778841	0.1458881
mRNA-1273	0.0220371	0.281305	0.08	0.938	-0.5293105	0.5733848
Ad26.COV2.S	-0.1233712	0.2452901	-0.5	0.615	-0.604131	0.3573886
MTX withdrawal (MTX-m)						
MTX-1ww	0.1800627	0.175424	1.03	0.305	-0.1637621	0.5238875
MTX-2ww	0.4886681	0.1783356	2.74	0.006	0.1391368	0.8381994
Constant	4.925829	0.3506851	14.05	0.000	4.238499	5.613159

Log S2 IFN-γ (SFU/10^6)	Coef.	Std. Err.	z	*p*	95% CI	
previous COVID-19	0.7797558	0.1690262	4.61	0.000	0.4484705	1.111041
Peptide batch						
2	0.2064351	0.2217905	0.93	0.352	-0.2282662	0.6411364
3	0.3729812	0.2323371	1.61	0.108	-0.0823911	0.8283535
4	0.7346361	0.2420884	3.03	0.002	0.2601515	1.209121
5	0.6386976	0.2329358	2.74	0.006	0.1821518	1.095243
6	0.3730285	0.2632834	1.42	0.157	-0.1429975	0.8890545
Ethnicity (White)						
Latin American	0.3186694	0.2157816	1.48	0.14	-0.1042547	0.7415936
Black	-1.389197	0.931566	-1.49	0.136	-3.215033	0.4366386
North African	1.304414	0.9299783	1.4	0.161	-0.5183095	3.127138
DAS28	-0.1272606	0.0771484	-1.65	0.099	-0.2784687	0.0239475
Lymphocyte count	-0.0001486	0.0000952	-1.56	0.119	-0.0003352	0.0000381
MTX dose	0.026391	0.0153345	1.72	0.085	-0.0036641	0.0564462
Vaccine type (BNT162b2)						
ChAdOX-1-S	-0.4285061	0.2207575	-1.94	0.052	-0.8611829	0.0041708
mRNA-1273	0.2126135	0.2645716	0.8	0.422	-0.3059373	0.7311642
Ad26.COV2.S	-0.5218369	0.2307032	-2.26	0.024	-0.974007	-0.0696669
MTX withdrawal (MTX-m)						
MTX-1ww	0.3666395	0.1641614	2.23	0.026	0.044889	0.6883901
MTX-2ww	0.5538222	0.1668972	3.32	0.001	0.2267097	0.8809347
Constant	4.664801	0.3787736	12.32	0.000	3.922418	5.407184

Sqrt RBD IFN-γ (SFU/10^6)	Coef.	Std. Err.	z	*p*	95% CI	
Sex						
Female	-1.760328	1.278378	-1.38	0.169	-4.265902	0.7452454
previous COVID-19	6.780517	1.21904	5.56	0.000	4.391242	9.169793
Peptide batch						
2	2.654986	1.618922	1.64	0.101	-0.5180433	5.828016
3	2.411654	1.722428	1.4	0.161	-0.9642419	5.78755
4	4.135419	1.798212	2.3	0.021	0.610988	7.65985
5	3.737758	1.682688	2.22	0.026	0.4397503	7.035765
6	3.359846	1.914488	1.75	0.079	-0.3924827	7.112174
Smoker						
Active	-1.054251	1.570146	-0.67	0.502	-4.13168	2.023177
Former	-1.576871	1.12357	-1.4	0.16	-3.779027	0.6252858
DAS28	-0.8449984	0.5655302	-1.49	0.135	-1.953417	0.2634204
Lymphocyte count	-0.0015099	0.0007055	-2.14	0.032	-0.0028927	-0.0001271
Vaccine type (BNT162b2)						
ChAdOX-1-S	-1.199217	1.63221	-0.73	0.463	-4.398291	1.999857
mRNA-1273	-0.0118651	1.918266	-0.01	0.995	-3.771597	3.747866
Ad26.COV2.S	-0.3442031	1.681216	-0.2	0.838	-3.639327	2.95092
MTX withdrawal (MTX-m)						
MTX-1ww	-0.6232148	1.217129	-0.51	0.609	-3.008744	1.762314
MTX-2ww	2.170701	1.206885	1.8	0.072	-0.1947516	4.536153
Constant	12.90248	2.498702	5.16	0.000	8.005116	17.79985

Log, logarithm base 10; sqrt, square root; *Coef, β-coefficient; std.err, standard error; CI, confidence interval*. Final generalised linear models after backward stepwise removal of variables with no influence in the model. *p*<0.05 was considered statistically significant.

Supplementary table 5. Multivariable analyses of IL-21 response.

Sqrt S1 IL21 (SFU/10^6)	Coef.	Std. Err.	z	*p*	95% CI	
Sex						
Female	0.4501544	0.4523703	1	0.32	-0.436475	1.336784
previous COVID-19	-0.5848048	0.4367321	-1.34	0.181	-1.440784	0.2711744
Peptide batch						
2	0.4268843	0.601418	0.71	0.478	-0.7518733	1.605642
3	0.4432819	0.6040534	0.73	0.463	-0.740641	1.627205
4	0.4692339	0.6125244	0.77	0.444	-0.7312918	1.66976
5	0.0479162	0.6144651	0.08	0.938	-1.156413	1.252246
6	1.529762	0.7121414	2.15	0.032	0.1339904	2.925533
DAS28	-0.2741072	0.2006579	-1.37	0.172	-0.6673896	0.1191751
Vaccine type (BNT162b2)						
ChAdOX-1-S	-0.230337	0.563331	-0.41	0.683	-1.334446	0.8737715
mRNA-1273	3.316077	0.7262979	4.57	0.000	1.892559	4.739594
Ad26.COV2.S	-0.4387918	0.6410937	-0.68	0.494	-1.695312	0.8177289
MTX withdrawal (MTX-m)						
MTX-1ww	-0.0559299	0.4378119	-0.13	0.898	-0.9140255	0.8021656
MTX-2ww	-0.0103141	0.4294069	-0.02	0.981	-0.8519361	0.831308
Constant	3.423244	0.7928554	4.32	0.000	1.869276	4.977212

Sqrt S2 IL21 (SFU/10^6)	Coef.	Std. Err.	z	*p*	95% CI	
Age	0.0313173	0.0165298	1.89	0.058	-0.0010804	0.0637151
previous COVID-19	-0.3783363	0.4269481	-0.89	0.376	-1.215139	0.4584666
Peptide batch						
2	0.2173451	0.5886499	0.37	0.712	-0.9363875	1.371078
3	-0.1148057	0.610266	-0.19	0.851	-1.310905	1.081294
4	0.5935514	0.6538793	0.91	0.364	-0.6880284	1.875131
5	-0.190893	0.6791914	-0.28	0.779	-1.522084	1.140298
6	2.219199	0.7918949	2.8	0.005	0.6671132	3.771284
DAS28	-0.3064896	0.1981438	-1.55	0.122	-0.6948443	0.0818651
MTX dose	0.0735837	0.0396894	1.85	0.064	-0.0042061	0.1513734
Days after vaccination	-0.0221359	0.0130357	-1.7	0.089	-0.0476854	0.0034135
Vaccine type (BNT162b2)						
ChAdOX-1-S	-1.02841	0.5440667	-1.89	0.059	-2.094761	0.037941
mRNA-1273	3.641483	0.7122493	5.11	0	2.2455	5.037466
Ad26.COV2.S	-0.8106379	0.6309292	-1.28	0.199	-2.047236	0.4259607
MTX withdrawal (MTX-m)						
MTX-1ww	-0.1470058	0.4288925	-0.34	0.732	-0.9876197	0.6936082
MTX-2ww	-0.52463	0.4200626	-1.25	0.212	-1.347938	0.2986777
Constant	2.332589	1.52035	1.53	0.125	-0.6472421	5.312421

RBD IL21 (SFU/10^6)	Coef.	Std. Err.	z	*p*	95% CI	
previous COVID-19	0.6400338	2.387412	0.27	0.789	-4.039207	5.319275
Peptide batch						
2	-0.4656361	3.3574	-0.14	0.89	-7.046019	6.114747
3	-0.745883	3.456693	-0.22	0.829	-7.520877	6.029112
4	2.66174	3.4488	0.77	0.44	-4.097783	9.421264
5	-3.202281	3.39482	-0.94	0.346	-9.856006	3.451444
6	5.529741	4.088649	1.35	0.176	-2.483864	13.54335
Comorbidities	-3.616277	2.108912	-1.71	0.086	-7.74967	0.5171148
Lymphocyte count	-0.002023	0.0013195	-1.53	0.125	-0.0046091	0.0005631
MTX dose	0.4989056	0.2218042	2.25	0.024	0.0641773	0.9336339
Days after vaccination	-0.0968901	0.07307	-1.33	0.185	-0.2401046	0.0463244
MTX withdrawal (MTX-m)						
MTX-1ww	-0.9571214	2.397268	-0.4	0.69	-5.655681	3.741438
MTX-2ww	2.429272	2.409025	1.01	0.313	-2.292331	7.150874
Constant	10.65643	5.606455	1.9	0.057	-0.3320212	21.64488

Sqrt, square root; *Coef, β-coefficient; std.err, standard error; CI, confidence interval*. Final generalised linear models after backward stepwise removal of variables with no influence in the model. $p<0.05$ was considered statistically significant.

Supplementary table 6. Cellular response and percentage of responders after vaccination.

	MTX-m (n=72)		MTX-1ww (n=71)		MTX-2ww (n=73)	
	No COVID-19 (n=51)	COVID-19 (n=21)	No COVID-19 (n=60)	COVID-19 (n=11)	No COVID-19 (n=58)	COVID-19 (n=15)
**S1 IFN-γ (SFU/10^6)**	115 (57.5-222.5)	362.5 (230-1055)	150 (80-310)	355 (90-690)	160 (80-365)	527.5 (260-715)
**Responders**	37 (72.5)	21 (100)	53 (88.3)	10 (90.9)	51 (87.9)	14 (93.3)
**Poor**	24 (47.1)	2 (9.5)	21 (35)	3 (27.3)	22 (37.9)	1 (6.7)
**Moderate**	13 (25.5)	2 (9.5)	16 (26.7)	1 (9.1)	10 (17.2)	2 (13.3)
**Good**	14 (27.5)	17 (81)	23 (38.3)	7 (63.6)	26 (44.8)	12 (80)
**S2 IFN-γ (SFU/10^6)**	120 (62.5-212.5)	270 (135-560)	215 (85-365)	275 (135-785)	220 (115-375)	392.5 (175-695)
**Responders**	39 (76.5)	20 (95.2)	50 (83.3)	11 (100)	51 (87.9)	15 (100)
**Poor**	26 (51)	5 (23.8)	24 (40)	3 (27.3)	15 (25.9)	2 (13.3)
**Moderate**	11 (21.6)	4 (19)	7 (11.7)	2 (18.2)	17 (29.3)	3 (20)
**Good**	14 (27.5)	12 (57.1)	29 (48.3)	6 (54.5)	26 (44.8)	10 (66.7)
**RBD IFN-γ (SFU/10^6)**	52.5 (25-80)	177.5 (90-400)	75 (40-110)	100 (40-185)	60 (35-170)	220 (65-345)
**Responders**	24 (47.1)	17 (81)	37 (61.7)	8 (72.7)	30 (51.7)	12 (80)
**Poor**	39 (76.5)	5 (23.8)	37 (61.7)	5 (45.5)	31 (53.4)	4 (26.7)
**Moderate**	3 (5.9)	3 (14.3)	12 (20)	2 (18.2)	6 (10.3)	1 (6.7)
**Good**	9 (17.6)	13 (61.9)	11 (18.3)	4 (36.4)	21 (36.2)	10 (66.7)
**S1 IL-21 (SFU/10^6)**	15 (0-30)	10 (5-20)	10 (5-27.5)	5 (0-15)	10 (5-25)	10 (5-20)
**Responders**	34 (66.7)	13 (61.9)	34 (56.7)	2 (18.2)	36 (62.1)	9 (60)
**Poor**	28 (54.9)	15 (71.4)	35 (58.3)	10 (90.9)	37 (63.8)	11 (73.3)
**Moderate**	3 (5.9)	1 (4.8)	5 (8.3)	1 (9.1)	6 (10.3)	1 (6.7)
**Good**	20 (39.2)	5 (23.8)	20 (33.3)	0 (0)	15 (25.9)	3 (20)
**S2 IL-21 (SFU/10^6)**	15 (5-35)	15 (10-20)	15 (5-25)	5 (0-20)	10 (5-45)	10 (0-20)
**Responders**	35 (68.6)	16 (76.2)	38 (63.3)	5 (45.5)	35 (60.3)	8 (53.3)
**Poor**	31 (60.8)	13 (61.9)	38 (63.3)	7 (63.6)	34 (58.6)	11 (73.3)
**Moderate**	2 (3.9)	3 (14.3)	4 (6.7)	3 (27.3)	4 (6.9)	2 (13.3)
**Good**	18 (35.3)	5 (23.8)	18 (30)	1 (9.1)	20 (34.5)	2 (13.3)
**RBD IL-21 (SFU/10^6)**	5 (0-10)	5 (0-10)	5 (0-10)	0 (0-5)	5 (0-10)	0 (0-20)
**Responders**	20 (39.2)	9 (42.9)	21 (35)	2 (18.2)	18 (31)	6 (40)
**Poor**	40 (78.4)	16 (76.2)	48 (80)	10 (90.9)	45 (77.6)	10 (66.7)
**Moderate**	6 (11.8)	1 (4.8)	4 (6.7)	1 (9.1)	3 (5.2)	1 (6.7)
**Good**	5 (9.8)	4 (19)	8 (13.3)	0 (0)	10 (17.2)	4 (26.7)

Estimated SFU/10^6 using the final multivariable models. All categorical variables are expressed as absolute count (percentage) and quantitative variables as median (IQR).

Supplementary table 7. Flare classification according to DAS28-CRP or physician criteria.

	MTX-m (n=72)	MTX-1ww (n=71)	MTX-2ww (n=73)	p-value
**DAS28 criteria**				
**No flare**	63	57	64	0.3
**Mild flare**	7	6	4	----
**Severe flare**	2	8	5	----
**Physician criteria**				
**No flare**	66	63	62	0.8
**Mild flare**	4	5	8	----
**Moderate flare**	2	3	3	----

	Physician criteria			
**DAS28 criteria**	**No flare**	**Mild flare**	**Moderate flare**	**p-value**
**No flare**	168	12	4	0.006
**Mild flare**	12	2	3	----
**Severe flare**	11	3	1	----

$p<0.05$ was considered statistically significant.

Supplementary table 8. Logistic regression analysis of factors associated with RA/PsA relapses.

RA/PsA relapse	OR	Std. Err.	z	*p*	95% CI	
Sex	0.7880935	0.4658336	-0.4	0.687	0.2474239	2.510232
Age	1.006718	0.017414	0.39	0.699	0.973159	1.041434
Rheumatoid factor	2.547234	1.383329	1.72	0.085	0.87863	7.384682
CRP at baseline	1.772955	0.4857022	2.09	0.037	1.036359	3.033087
Nodules	3.103978	2.24654	1.56	0.118	0.7513665	12.82288
Asthma	3.222932	2.160552	1.75	0.081	0.8662282	11.9914
MTX-1ww	1.926337	1.181906	1.07	0.285	0.5787307	6.411916
MTX-2ww	1.924575	1.128944	1.12	0.264	0.6095721	6.076372
Constant	0.0225483	0.0277201	-3.08	0.002	0.0020262	0.2509299

RA, rheumatoid arthritis; PsA, psoriatic arthritis; OR, odds ratio; Std.Err, standard error; CI, confidence interval; CRP, C-reactive protein. $p<0.05$ was considered statistically significant.

Supplementary table 9. Number of individuals with one or two vaccine doses, type of administered vaccine, and interdose period between doses.

	MTX-m (n=72)		MTX-1ww (n=71)		MTX-2ww (n=73)	
	No COVID-19 (n=51)	COVID-19 (n=21)	No COVID-19 (n=60)	COVID-19 (n=11)	No COVID-19 (n=58)	COVID-19 (n=15)
**One dose**						
**BNT162b2**	0 (0)	4 (57.1)	0 (0)	3 (50)	4 (40)	5 (83.3)
**ChAdOx-1-S**	0 (0)	1 (14.3)	0 (0)	0 (0)	0 (0)	0 (0)
**mRNA-1273**	0 (0)	0 (0)	0 (0)	1 (16.7)	1 (10)	0 (0)
**Ad26.COV2.S**	2 (100)	2 (28.6)	7 (100)	2 (33.3)	5 (50)	1 (16.7)
**Total**	2	7	7	6	10	6
**Two-dose**						
**BNT162b2**	38 (77.5)	9 (64.3)	41 (77.4)	3 (60)	39 (81.3)	7 (77.8)
**Interdose (days)**	21 (21-21)	21 (21-22)	21 (21-21)	21 (21-22)	21 (21-22)	21 (21-22)
**ChAdOx-1-S**	6 (12.2)	5 (35.7)	11 (20.8)	2 (40)	4 (8.3)	1 (11.1)
**Interdose (days)**	71 (70-77)	70 (69-77)	71 (66-77)	73.5 (65-82)	72 (68.5-76.5)	90
**mRNA-1273**	5 (10.2)	0 (0)	1 (1.9)	0 (0)	5 (10.4)	1 (11.1)
**Interdose (days)**	28 (28-30)	----	28	----	28 (28-28)	28
**Total**	49	14	53	5	48	9

All categorical variables are expressed as absolute count (percentage) and quantitative variables as median (IQR).

Supplementary table 10. Antibody and cellular response at baseline (pre-vaccination).

	MTX-m (n=72)	MTX-1ww (n=71)	MTX-2ww (n=73)
**anti-S IgG (ΔAU/mL)**	5.4 (2.6-76.9)	4.1 (3-9.3)	3.6 (2.6-6.8)
**anti-S IgA (ΔAU/mL)**	2.9 (1.2-12.2)	2.1 (1.2-4.8)	1.5 (0.9-4.6)
**anti-S IgM (ΔAU/mL)**	3 (1.5-10.3)	2.5 (1.7-4.7)	6.5 (4.7-9.3)
**anti-RBD IgG (ΔAU/mL)**	6.5 (4.7-9.3)	6.8 (4.4-8.7)	6.5 (4.1-8.9)
**anti-RBD IgA (ΔAU/mL)**	2.6 (1.9-3.7)	2.5 (1.9-3.9)	2.7 (1.6-3.5)
**anti-RBD IgM (ΔAU/mL)**	1.3 (0.8-2.5)	1.7 (1.2-2.6)	1.6 (1.1-3.1)
**S1 IFN-γ (SFU/10^6)**	25 (10-60)	20 (10-67.5)	20 (10-67.5)
**S2 IFN-γ (SFU/10^6)**	32.5 (15-82.5)	20 (10-57.5)	27.5 (10-70)
**RBD IFN-γ (SFU/10^6)**	20 (5-47.5)	15 (5-60)	15 (7.5-35)
**S1 IL-21 (SFU/10^6)**	0 (0-5)	0 (0-2.5)	0 (0-0)
**S2 IL-21 (SFU/10^6)**	0 (0-0)	0 (0-0)	0 (0-0)
**RBD IL-21 (SFU/10^6)**	0 (0-0)	0 (0-0)	0 (0-0)

Quantitative variable are expressed as median (IQR)
